# Promoter Hypermethylation and Suppression of Glutathione Peroxidase 3 Are Associated with Inflammatory Breast Carcinogenesis

**DOI:** 10.1155/2014/787195

**Published:** 2014-03-20

**Authors:** Mona M. Mohamed, Salwa Sabet, Dun-Fa Peng, M. Akram Nouh, Mohamed El-Shinawi, Wael El-Rifai

**Affiliations:** ^1^Department of Zoology, Faculty of Science, Cairo University, Giza 12613, Egypt; ^2^Department of Surgery, Vanderbilt University Medical Center, Nashville, TN 37232, USA; ^3^Department of Pathology, National Cancer Institute, Cairo University, Giza 12613, Egypt; ^4^Department of General Surgery, Faculty of Medicine, Ain Shams University, Cairo 11566, Egypt

## Abstract

Reactive oxygen species (ROS) play a crucial role in breast cancer initiation, promotion, and progression. Inhibition of antioxidant enzymes that remove ROS was found to accelerate cancer growth. Studies showed that inhibition of glutathione peroxidase-3 (GPX3) was associated with cancer progression. Although the role of GPX3 has been studied in different cancer types, its role in breast cancer and its epigenetic regulation have not yet been investigated. The aim of the present study was to investigate GPX3 expression and epigenetic regulation in carcinoma tissues of breast cancer patients' in comparison to normal breast tissues. Furthermore, we compared GPX3 level of expression and methylation status in aggressive phenotype inflammatory breast cancer (IBC) versus non-IBC invasive ductal carcinoma (IDC). We found that GPX3 mRNA and protein expression levels were downregulated in the carcinoma tissues of IBC compared to non-IBC. However, we did not detect significant correlation between GPX3 and patients' clinical-pathological prosperities. Promoter hypermethylation of GPX3 gene was detected in carcinoma tissues not normal breast tissues. In addition, IBC carcinoma tissues showed a significant increase in the promoter hypermethylation of GPX3 gene compared to non-IBC. Our results propose that downregulation of GPX3 in IBC may play a role in the disease progression.

## 1. Introduction

The breast tumor microenvironment is characterized by the release of endogenous reactive oxygen species (ROS) that resulted from accumulation of different metabolic and pathological changes such as glucose deprivation, steroid hormones metabolism by lactoperoxidase [1], mitochondrial disorder [2], infiltration of macrophages [3], and angiogenesis and reperfusion of blood vessels [4, 5]. Removal of ROS from tissues is achieved by the family of glutathione peroxidases (GPXs) (Enzyme Commission number 1.11.1.9) expressed cytoplasmically and their expression is tissue specific [6]. GPXs are known to protect cells against oxidative stress by catalyzing the reduction of H_2_O_2_, organic hydroperoxide and lipid peroxides by reduced glutathione [6].

Among the family of GPXs, the isoenzyme GPX3 is found to play a crucial role in the removal of ROS and healthy tissue detoxification [6]. In addition, studies showed that expression and activity of GPX3 contribute to prevention of cancer initiation [7, 8]. Paradoxically, GPX3 inhibition is suggested to be associated with different stages of cancer progression including initiation, promotion, and metastasis [6]. GPX3 was found to be downregulated in the plasma of breast, gastric, and colorectal cancer patients [9]. Moreover, GPX3 downregulation was reported in prostate cancer tissues [10], thyroid cancer [11], and esophageal cancer [12, 13]. Promoter hypermethylation mechanism which is a “frequent event in human cancers” may result in GPX3 gene silencing and inhibition of GPX3 expression [14]. GPX3 promoter hypermethylation and downregulation were detected in prostate cancer [15]; endometrial adenocarcinoma [16]; cervical, thyroid, and lung cancer [17]; head and neck carcinoma [14]; gastric cancer [18]; and multiple myeloma [19]. GPX3 hypermethylation correlates with disease poor prognosis and resistance to chemotherapy in head and neck cancer patients [14] and multiple myeloma [19]. Recently, we found that inactivation of the GPX3 gene by promoter hypermethylation in gastric cancer is associated with high incidence of lymph node metastasis [20].

Inflammatory breast cancer (IBC) is an aggressive and highly metastatic form of breast cancer, most prominent among premenopausal women [21]. IBC is characterized by rapid onset over a period of only weeks to a few months, and patients presented with erythema, edema of the breast, and a “peau d'orange” appearance of the skin [22, 23]. Almost all IBC patients had lymph node metastasis at time of diagnosis. Despite the distinct clinical features associated with IBC, the genetic and epigenetic signature underlining the aggressive metastatic behavior of IBC remains poorly understood [24]. Studies showed that in carcinoma tissues ROS induce cell proliferation, motility, invasion, angiogenesis, and the escape from apoptotic mechanism [25]. ROS was found to stimulate cancer cell motility and invasion by activating protein kinase-C (PKC) and the mitogen-activated protein kinase (MAPK) and extracellular signal-regulated kinases (ERKs) signaling pathways, thus increasing the risk of metastasis [26, 27]. Furthermore, we found that promoter methylation and loss in copy number of GPX3 gene are associated with the number of lymph node metastases in gastric cancer [20]. Since almost all IBC patients presented with positive axillary lymph node metastasis [21], herein, we investigated whether expression and epigenetic regulation of GPX3 may contribute to the aggressive phenotype IBC versus non-IBC.

## 2. Materials and Methods

### 2.1. Reagents

DNeasy tissue kit was purchased from Qiagen (Valencia, CA, USA). EZ DNA Methylation-Gold Kit was purchased from ZYMO Research (Orange, CA, USA). Dako Antibody Diluent-Reduce Background and Dako Cytomation EnVision+ Dual Link System-HRP (DAB+) kit (catalog number K4065) were purchased from Dako (Carpinteria, CA, USA). Mouse anti-GPX3 monoclonal antibody, Clone 23B1, was purchased from Abcam, Cambridge, MA, USA. Permount was purchased from Fisher Scientific (Fair Lawn, NJ, USA). Unless otherwise stated all other reagents were from Sigma (St. Louis, MO, USA).

### 2.2. Patients and Tissues

Patients were enrolled from Breast Clinics of Ain Shams University Hospitals, Ain Shams University, Cairo, Egypt, after obtaining the Institutional Review Board (IRB) approval from the ethics committee of Ain Shams University. All patients signed informed consent before participating in the study. Breast cancer diagnosis was achieved by clinical examination, ultrasound, mammography, and biopsy. In the present study, we enrolled 40 women clinically diagnosed with breast cancer. Patients were divided into non-IBC (*n* = 20) and IBC (*n* = 20) subgroups. Patients were considered to harbor IBC when they presented with a swollen breast, skin inflammation, and edema as previously described [21]. In addition, 6 normal breast tissue samples donated by healthy volunteers undergoing mammoplasty were used as control.

Tissue samples were collected either from preadjuvant chemotherapy tissue biopsies or during modified radical mastectomy. Tissue samples were divided into two pieces, one snap-frozen at −80°C in RNAlater (Qiagen) and one fixed in 10% neutral buffered formalin and processed for sectioning for routine pathological examination as well as for immunohistochemistry. Pathological data regarding tumor size, tumor grade [28], presence and absence of lymphovascular invasion, and dermal and tumor stromal lymphatic emboli were assessed for routine diagnosis as we described before [21].

### 2.3. Assessment of mRNA Expression of GPX3 Using Quantitative Real-Time Polymerase Chain Reaction (qRT-PCR)

We isolated total RNA from frozen breast tissue samples using an RNeasy Mini kit (Qiagen), following the manufactory's instructions. Single-stranded cDNA was synthesized using the iScript cDNA Synthesis Kit (Bio-Rad, Hercules, CA, USA). We assessed transcription levels of GPX3 in breast cancer samples versus normal tissues (control) from breast of healthy volunteers using qRT-PCR. GPX3 primers were used as we described before (forward 5′-GCCGGGGACAAGAGAAGT-3′ and reverse 5′-GAGGACGTATTTGCCAGCAT-3′) [20]. Quantitative real-time PCR (qRT-PCR) reactions were carried out using Bio-Rad IQ SYBR GREEN Supermix (Bio-Rad, CA, USA) on iCycler (Bio-Rad, CA, USA), with the threshold cycle number determined by use of iCycler software version 3.0. All samples were run in triplicate, and the threshold cycle (Ct) was determined using the iCycler software and then was averaged. Results were normalized to internal control* Hypoxanthine phosphoribosyltransferase-1* (HPRT1) and GPX3 expression fold was calculated according to the formula 2(*R*_*t*_ − *E*_*t*_)/2(*R*_*n*_ − *E*_*n*_) as previously described [29], where *R*_*t*_ is the threshold cycle number for the reference gene observed in the tumor, *E*_*t*_ is the threshold cycle number for the experimental gene observed in the tumor, *R*_*n*_ is the threshold cycle number for the reference gene observed in the normal samples, and *E*_*n*_ is the threshold cycle number for the reference gene observed in the tumor. *R*_*n*_ and *E*_*n*_ values were calculated as an average of the 6 normal samples. For all primary BACs, the gene was considered to be downregulated if the mRNA expression fold was ≤0.5 in comparison with the normal samples [20].

### 2.4. Immunohistochemistry

Tissue sections of 5 *μ*m thickness were prepared from paraffin blocks of breast carcinoma and normal breast tissues and stained with hematoxylin and eosin to select sections suitable for immunostaining and scoring. Tissue sections were first deparaffinized and rehydrated through graded concentrations of ethanol. For antigen retrieval, slides were incubated in citrate buffer (pH 6.0) in a water bath for 1 h at 99°C. Slides were cooled by incubation in Tris-buffered saline (TBS: 0.05 mol/L Tris-HCl, pH 7.6, 0.15 mol/L NaCl, and 0.05% Tween 20) for 20 min. Endogenous peroxidase activity was blocked by using Dako Dual Endogenous Enzyme Block for 10 min. For immunohistochemical stain of GPX3, tissue sections were incubated for 1 h at room temperature with the primary antibody mouse anti-GPX3 (mouse anti-GPX3 monoclonal antibody, Clone 23B1, Abcam). The monoclonal antibody was diluted 1 : 100 in Dako Antibody Diluent-Reduce Background product (DAKO). Detection was carried out by incubating sections with 100 *μ*L of Horseradish Peroxidase (HRP) Rabbit/Mouse (EnVision+ Dual Link System-HRP diaminobenzidine (DAB+)) for 45 min. Staining was achieved by adding 100 *μ*L of DAB+ diluted 1 : 50 in substrate buffer [EnVision+ Dual Link System-HRP (DAB+)]. Staining was progressed for 15 min until the development of brown color. Nuclei were counterstained with hematoxylin, rinsed in PBS, and mounted using Permount for microscopic examination. Negative control slides were run in parallel with each marker where the primary antibody is replaced by PBS. The level of expression of GPX3 protein was scored according to both the intensity and the positivity of the stain of the cells within the entire slide: negative, no immunostaining is observed; score (+), less than 10% of cells showed no or weak staining; score (++), 10–50% of cells showed moderate to strong staining; and score (+++), more than 50% of cells showed strong staining [21].

### 2.5. DNA Extraction from Fresh Tissue and Identification of CpG Islands in the Promoter Region of GPX3 Gene

Genomic DNA was extracted from 25 mg of fresh tissue of biopsy or modified radical mastectomy using DNeasy tissue kit Qiagen. In the last step of the protocol, DNA was eluted in 200 *μ*L elution buffer. We used University of California's (UCSC) Genome Browser website (http://genome.ucsc.edu/) to obtain DNA sequences around the promoter region. Identified sequences were confirmed to be identical to that from the DBTSS (database of transcriptional start sites, http://dbtss.hgc.jp/) as described [29]. The CpG island in the promoter region of GPX3 was defined using CpG island searcher online tool (http://www.uscnorris.com/cpgislands2/cpg.aspx).

### 2.6. DNA Bisulfite Treatment and Methylation-Specific PCR (MSP)

Bisulfite modification of the purified DNA was achieved by using an EZ DNA methylation-Gold kit (ZYMO Research, Orange, CA, USA) following the manufactory's instructions. We used 1 *μ*g of extracted genomic DNA of each sample from normal breast tissue samples, IBC and non-IBC breast carcinoma tissue samples [12, 29]. The bisulfite treated DNA was subjected to MSP in a final reaction of 50 *μ*L. We designed primers for MSP targeting CpG-rich promoter region using online software “MethPrimer” (http://www.urogene.org/methprimer/). The forward and reverse primers used for methylated GPX3 were 5′-GTTGAGGGTAAGTCGCGTTC-3′ and 5′-GTCCGTCTAAAATATCCGACG-3′ and those for unmethylated GPX3 were 5′-GAGTTGAGGGTAAGTTGTGTTTGT-3′ and 5′-CCATCCATCTAAAATATCCAACACT-3′. PCR mixture included the Platinum PCR SuperMix High Fidelity (Invitrogen, Carlsbad, CA, USA) and the program was adjusted as follows: initial denaturation at 94°C for 5 min, then 35 cycles consisting each of 94°C for 1 min, annealing at 54°C for 1 min, and finally extension at 72°C for 1 min. PCR products were subjected to electrophoresis on 1.5% agarose gel using GelRed nucleic acid stain (Biotium, Hayward, CA, USA) and amplified DNA was visualized by 300 nm transillumination. For quantitative analysis, visualized bands of the agarose gel were analyzed by ImageJ (National Institutes of Health, Bethesda, MA, USA) software. For each carcinoma tissue specimen, the band intensity of the methylated and unmethylated MSP products was quantified and normalized against gel background as described elsewhere [30].

### 2.7. Statistical Analysis

The data was analyzed using SPSS software version 16.0. Differences were evaluated by Student's *t*-test and Fisher's exact test. *P* < 0.05 was considered as statistically significant.

## 3. Results

### 3.1. Clinical and Pathological Features of IBC and Non-IBC Patients

The clinical and pathological features of non-IBC and IBC patients are presented in Table 1. All of IBC patients were premenopausal one decade younger than non-IBC patients (*P* < 0.001). Statistical analysis revealed that IBC patients showed a significant higher incidence (*P* < 0.01) of positive metastatic lymph nodes compared to non-IBC patients. In addition, a significant difference (*P* < 0.001) in lymphovascular invasion and dermal lymphatic emboli was highly detected in IBC versus non-IBC patients tissue sections.

### 3.2. Downregulation of the GPX3 Protein Expression in Breast Carcinoma Tissue

**Figure 1:**
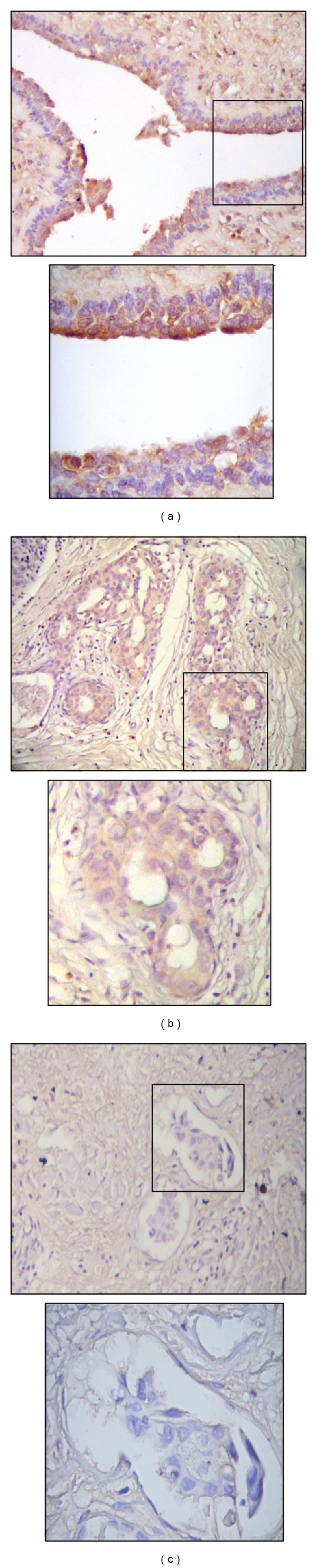
GPX3 protein is downregulated in IBC. Microscopic images representative of IHC stain of GPX3 (brown color) in (a) normal breast tissues showing moderate to marked intensity of GPX3, (b) non-IBC tissue sections showing mild intensity of GPX3, and (c) IBC tissue sections with no immunostaining of GPX3 by carcinoma cells within tumor emboli (magnification: upper panel, 10x; lower panel, 40x).

Level of expression of GPX3 protein was assessed in normal and carcinoma breast tissues using IHC. GPX3 expression staining results were scored for the positivity and intensity (Table 2). Statistical analysis, using the Chi-square test, revealed that normal breast tissues express significantly high level of GPX3 protein (Figure 1(a)) compared to breast carcinoma tissues of non-IBC (Figure 1(b)) and IBC (Figure 1(c)) patients (*P* = 0.001 and *P* < 0.001, resp.). In addition, non-IBC tissues showed a significant increase (*P* = 0.043) in the level of expression of GPX3 as compared to those of IBC patients.

### 3.3. Suppression of GPX3 mRNA Expression in IBC Carcinoma Tissue Samples

**Figure 2:**
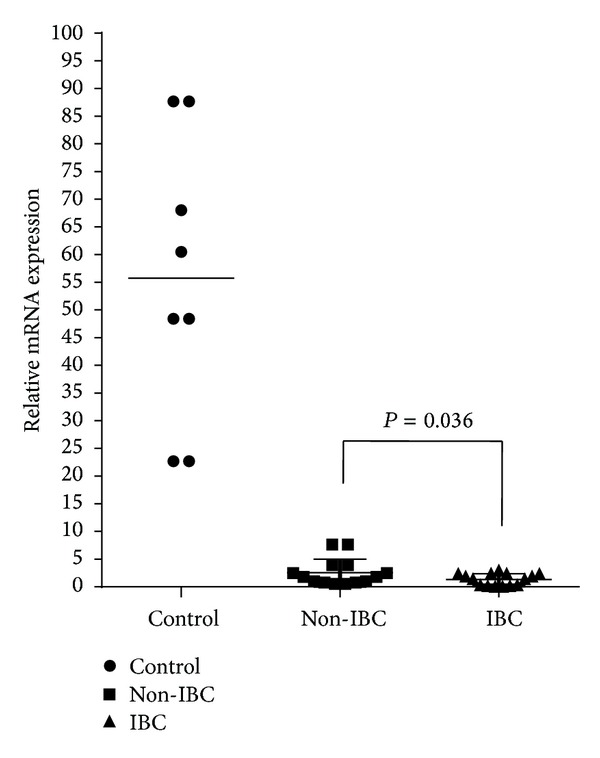
Expression of GPX3 mRNA is downregulated in IBC. Blot represents the mean fold change of GPX3 mRNA measured by RT-PCR in normal breast tissues, IBC and non-IBC carcinoma tissues. Statistical analysis revealed a significant increase in expression of GPX3 mRNA in normal breast tissues compared to non-IBC and IBC carcinoma tissues (*P* = 0.001 and *P* < 0.001, resp.). In carcinoma tissues of non-IBC patients, the level of expression of GPX3 mRNA is significantly higher (*P* = 0.036) than that of IBC. Results are representative of at least three independent experiments. Data are expressed as mean ± SD and *P* value was determined by Student's *t*-test.

Inhibition of GPX3 protein expression in the carcinoma tissues of breast cancer patients was confirmed at mRNA level by using qRT-PCR. We found that the level of GPX3 mRNA expression in normal breast tissues was significantly higher than that in breast carcinoma tissue samples of non-IBC and IBC (*P* = 0.001 and *P* < 0.001, resp.). We compared the level of mRNA expression of GPX3 in non-IBC and IBC, and our results revealed that mRNA expression level of GPX3 expression in non-IBC carcinoma tissues was statistically significant (*P* = 0.036) higher than that in IBC (Figure 2). Using the Pearson correlation statistical analysis, we did not detect significant correlation between downregulation of GPX3 mRNA and patients clinical-pathological properties (tumor size, tumor grade, and number of axillary metastatic lymph nodes) in non-IBC and IBC patients. However, downregulation of GPX3 in IBC versus non-IBC carcinoma tissues suggests that GPX3 may play a role in IBC disease progression.

### 3.4. Methylation Profile of GPX3 Promoter Region in Breast Cancer Tissue Samples

**Figure 3:**
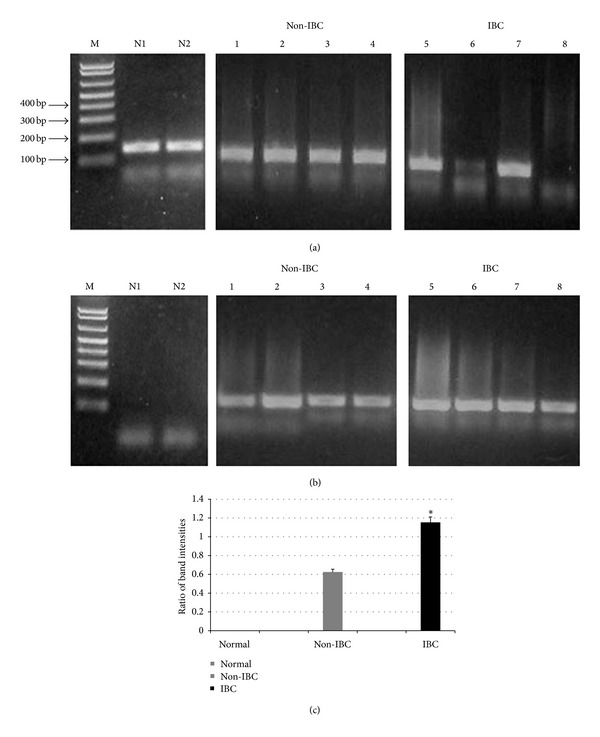
Gel electrophoresis of GPX3 methylation-specific PCR (GPX3-MSP) products. Representative results of MSP using (a) unmethylated primers and (b) methylated primers. M is the DNA marker; lanes N1 and N2 represent normal breast tissues, lanes 1–4 represent non-IBC carcinoma tissues, and lanes 4–8 represent IBC breast carcinoma tissues. (c) Bars represent intensity values of methylated/unmethylated (M/U) ratios as quantified by ImageJ software. Normal breast tissues (*n* = 6) with an M/U ratio = 0 (no methylated bands were detected from MSP) were recognized as unmethylated. We detected a significant increase (*P* = 0.04) in the M/U ratio in IBC (*n* = 20) carcinoma tissues compared to non-IBC (*n* = 20) carcinoma tissues.

Analysis of the promoter region of GPX3 indicated that CpG islands were present within −1000 to +300 bp of the gene. We assessed the GPX3 promoter region methylation in 40 breast cancer tissue samples and 6 normal breast tissue samples. Agarose gel electrophoresis of all normal breast tissues showed only one band corresponding to unmethylated GPX3-MSP products (Figure 3(a)). On the contrary, agarose gel electrophoresis of all GPX3-MSP products breast carcinoma tissue samples showed bands of 200 bp corresponding to both unmethylated (Figure 3(a)) and methylated GPX3-MSP products (Figure 3(b)). The present results showed that GPX3 methylation was detected in breast carcinoma tissues and not in normal breast tissues. Thus, GPX3 promoter methylation is responsible for the downregulation of GPX3 mRNA in breast carcinoma tissues since it was not detected in normal breast tissues. It should be noted that detection of methylated and unmethylated products in some breast carcinoma tissue homogenates may be due to heterogeneous population of breast carcinoma cells and/or the presence of other normal stromal cells from breast tumor microenvironment.

For quantitative analysis, visualized bands of agarose gel were analyzed by ImageJ (National Institutes of Health, Bethesda, MA, USA) software. For each carcinoma tissue specimen, the band intensity of the methylated and unmethylated GPX3-MSP products was quantified and normalized against gel background as described elsewhere [30]. Intensity values of methylated/unmethylated (M/U) ratio for GPX3-MSP products for non-IBC (*n* = 20) and IBC (*n* = 20) patients were analyzed by Student's *t*-test. Normal breast tissues (N) with an M/U ratio = 0 (no methylated bands were detected from MSP) were assessed as unmethylated. Results revealed a significant increase (*P* = 0.04) in the M/U ratios of GPX3-MSP products in IBC versus non-IBC carcinoma tissues (Figure 3(c)). Significant increase in M/U ratio in IBC versus non-IBC suggests a potential role for GPX3 in IBC disease progression.

## 4. Discussion

Elevated levels of ROS detected in breast cancer were found to play a crucial role in the disease progression [4]. For instance, carcinoma cells utilize ROS to stimulate cancer cell proliferation, motility, invasion, angiogenesis, and escape of apoptotic mechanism [25]. In addition, ROS augment carcinoma cell motility and invasion by activating protein kinase-C (PKC) and the ERK/MAPK signaling pathways, thus increasing the risk of metastasis [26, 27]. Failure to remove exogenous and endogenous ROS may occur due to defect in the cellular antioxidant system of carcinoma cells represented by the inhibition of the activity of antioxidant enzymes as GPXs family members. Thus, drugs which induce expression of antioxidant enzymes were suggested for cancer treatment [29]. GPXs are categorized into two types; one type is selenium-dependent catalytic activity (GPX-1, -2, -3, -4, and 6) and the second type is non-selenium-dependent (GPX-5 and -7) [6]. Downregulation and genetic imbalance among GPXs were found to play a key role in breast cancer. For example, GPX1 gene allelic variants and loss of heterozygosity (LOH) at 3p21.3p region contribute to breast cancer development [31]. Low expression of GPX4 in breast-invasive ductal carcinoma correlated with high tumor grade and poor prognosis of breast cancer patients [32]. Low expression of GPX3 significantly correlates with high risk of breast cancer local recurrence among early-stage invasive breast cancer patients, regardless of patients' clinic-pathological criteria [33].

In fact, GPX3 is an essential enzyme responsible for the removal of ROS in healthy tissues. On the contrary, GPX3 was found to be downregulated in carcinoma tissues of breast, gastric, and colorectal cancer patients [9], prostate cancer [10], thyroid cancer [11], and esophageal cancer [12]. The impaired function of GPX3 would result in the accumulation of an increased amount of hydrogen peroxide and other ROS which may induce breast carcinogenesis via induction of oxidative DNA damage, genetic instability, neoplastic transformation [34], and mutation of the p53 tumor suppressor gene [35]. Epigenetic mechanisms such as DNA hypermethylation and histone modification may repress the expression of GPX3. For instance, treatment of SKGT4 esophageal cancer cell lines [29] and endometrial tumor cell lines (NUT12 and NUT81) [16], that show GPX3 promoter hypermethylation, with the demethylating agent 5-aza-2′-deoxycytidine (5Aza-dC) and the histone deacetylace inhibitor, trichostatin A (TSA) results in re-expression of GPX3 mRNA. These results suggest that DNA hypermethylation and histone deacetylation may act together to regulate the expression of GPX3 mRNA. Paradoxically, downregulation of GPX3 in absence of promoter hypermethylation is associated with GPX3 gene deletion in endometrial tumor cell line (NUT84) [16]. In fact, GPX3 promoter hypermethylation is linked to downregulation of GPX3 expression in different types of cancer cells and treatment with 5-Aza to human esophageal adenocarcinoma cancer cells SKGT4 [29] and human myeloma cells KMS11 [19] restores GPX3 gene expression.

Our previous studies demonstrated that GPX3 was downregulated in Barrett's carcinoma due to hypermethylation of the promoter region [12]. In addition, we found that loss in DNA copy number, hypermethylation of the promoter region, and downregulation of mRNA expression of GPX3 are associated with lymph node metastasis in gastric carcinomas. Reactivation of GPX3 in gastric adenocarcinoma cell line AGS inhibits cell motility as assessed by wound healing assay [20]. Herein, we studied the role of GPX3 in breast carcinogenesis. We analyzed the level of expression of GPX3 in normal breast tissues obtained from healthy volunteers during mammoplasty, and non-IBC and IBC breast carcinoma tissues. Our results revealed that GPX3 protein level and mRNA were significantly expressed in normal breast tissues and downregulated in breast carcinoma tissues. The present results are consistent with other studies which proved that GPX3 is downregulated in carcinoma tissues such as prostate [10], thyroid [11], and esophageal [12]. When we compared carcinoma tissues of IBC with non-IBC samples, we detected a significant decrease in mRNA and protein expression of GPX3 in IBC tissue samples versus non-IBC tissue samples. We did not detect any significant correlation between downregulation of GPX3 mRNA and patients clinical-pathological properties. The present results agree with other studies that showed downregulation of GPX3 in endometrial adenocarcinoma [16] and early invasive breast carcinoma [33] regardless of patients' clinical and pathological criteria.

IBC is an aggressive phenotype, characterized by high metastatic potential, disease recurrence, and resistance to chemotherapy [36]. Furthermore, recently we found that IBC carcinoma tissues are characterized by high infiltration of tumor associated macrophages that enhance carcinoma cells invasion and motility [37]. Interestingly, loss of GPX3 contributes to high infiltration of tumor associated macrophages that support tumor survival in GPX3 knockout mice model [38]. Thus, inhibition of GPX3 in IBC carcinoma tissues may be associated with the high infiltration of macrophages. In addition, quantitative analysis of the band intensities of the corresponding methylated/unmethylated MSP products revealed a significant increase in GPX3 promoter hypermethylation in IBC carcinoma tissues versus non-IBC carcinoma tissues. We assumed that GPX3 may contribute to IBC molecular phenotype.

## 5. Conclusion

Our results suggest that epigenetic regulation of GPX3 occurred widely in breast cancer tissues compared to normal breast tissues and this may be due to GPX3 promoter hypermethylation in breast cancer cells and not in normal breast tissues. In addition, methylation silencing of GPX3 in IBC may contribute to invasion of IBC carcinoma cells into lymphatic vessels, formation of tumor emboli, and IBC chemoresistance as suggested in other cancers [14, 19]. Further studies to validate the role of GPX3 as a prognostic maker in IBC and identify the mechanisms by which GPX3 is involved in IBC carcinogenesis are essential.

## Figures and Tables

**Table 1:** Patients' clinical and pathological data.

Characteristics	Non-IBC *n* = 20 (%)	IBC *n* = 20 (%)	*P* value
Age			
Mean ± SE	54.35 ± 2.1	40.55 ± 1.7	
≤50	7 (35)	18 (90)	<0.001*
>50	13 (65)	2 (10)	
Tumor size**			
Mean ± SE	5.12 ± 0.5	7.08 ± 0.6	
≤2 cm	1 (5)	0	>0.05
>2 cm	19 (95)	18 (90)	
Tumor grade			
G2	15 (75)	14 (70)	>0.05
G3	5 (25)	6 (30)	
Lymph node status**			
Positive	17 (85)	18 (90)	>0.05
Negative	3 (15)	0	
Number of lymph nodes**			
≤6	16 (80)	6 (33.3)	<0.01*
>6	4 (20)	12 (66.7)	
Lymphovascular invasion			
Positive	2 (10)	14 (70)	<0.001*
Negative	18 (90)	6 (30)	
Dermal lymphatic emboli			
Positive	2 (10)	18 (90)	<0.001*
Negative	18 (90)	2 (10)	
ER status			
Positive	7 (35)	6 (30)	>0.05
Negative	13 (65)	14 (70)	
PR status			
Positive	9 (45)	7 (35)	>0.05
Negative	11 (55)	13 (65)	
HER-2 status			
Positive	2 (10)	4 (20)	>0.05
Negative	18 (90)	16 (80)	

*Significant *P* value calculated by Student's *t*-test.

**n = 18 in IBC patients.

**Table 2:** Scoring of GPX3 expression in normal, non-IBC, and IBC breast tissues.

Score	Normal (*n* = 6)	Non-IBC (*n* = 16)^a^	IBC (*n* = 16)^b,c^
	*n* (%)	*n* (≈%)	*n* (≈%)
Negative	0 (0%)	5 (31.2%)	11 (68.7%)
+	0 (0%)	7 (43.8%)	3 (18.7%)
++	3 (50%)	4 (25%)	2 (12.5%)
+++	3 (50%)	0	0

Significant *P* value calculated by the Chi-square test.

^a^Significant *P* value (*P* = 0.001) when normal tissues were compared to non-IBC tissues.

^b^Significant *P* value (*P* < 0.001) when normal tissues were compared to IBC tissues.

^c^Significant *P* value (*P* = 0.043) when non-IBC tissues were compared to IBC tissues.

*n* = number of patients.
